# Role of Linker Functionality in Polymers Exhibiting Main‐Chain Thermally Activated Delayed Fluorescence

**DOI:** 10.1002/advs.202200056

**Published:** 2022-03-06

**Authors:** Kai Philipps, Yutaka Ie, Bas van der Zee, Rui‐Qi Png, Peter K. H. Ho, Lay‐Lay Chua, Esther del Pino Rosendo, Charusheela Ramanan, Gert‐Jan A. H. Wetzelaer, Paul W. M. Blom, Jasper J. Michels

**Affiliations:** ^1^ Max Planck Institute for Polymer Research Ackermannweg 10 55128 Mainz Germany; ^2^ The Institute of Scientific and Industrial Research (ISIR) Osaka University 8‐1 Mihogaoka Ibaraki Osaka 567‐0047 Japan; ^3^ Department of Physics National University of Singapore Lower Kent Ridge Road Singapore S117550 Singapore; ^4^ Department of Physics National University of Singapore Lower Kent Ridge Road Singapore S117552 Singapore

**Keywords:** high efficiency, organic light‐emitting diodes, thermally activated delayed fluorescence

## Abstract

Excellent performance has been reported for organic light‐emitting diodes (OLEDs) based on small molecule emitters that exhibit thermally activated delayed fluorescence. However, the necessary vacuum processing makes the fabrication of large‐area devices based on these emitters cumbersome and expensive. Here, the authors present high performance OLEDs, based on novel, TADF polymers that can be readily processed from a solution. These polymers are based on the acridine‐benzophenone donor–acceptor motif as main‐chain TADF chromophores, linked by various conjugated and non‐conjugated spacer moieties. The authors’ extensive spectroscopic and electronic analysis shows that in particular in case of alkyl spacers, the properties and performance of the monomeric TADF chromophores are virtually left unaffected by the polymerization. They present efficient solution‐processed OLEDs based on these TADF polymers, diluted in oligostyrene as a host. The devices based on the alkyl spacer‐based TADF polymers exhibit external quantum efficiencies (EQEs) ≈12%, without any outcoupling‐enhancing measures. What's more, the EQE of these devices does not drop substantially upon diluting the polymer down to only ten weight percent of active material. In contrast, the EQE of devices based on the monomeric chromophore show significant losses upon dilution due to loss of charge percolation.

## Graphical abstract

The authors present novel solution‐processable polymer emitters that exhibit thermally activated delayed fluorescence (TADF). The polymers comprise acrydine‐benozphenone chromophores, linked by various non‐conjugated and conjugated spacer moieties. In case of alkyl spacers, the emitting properties of the TADF‐chromophores are unaffected by the polymerization. Organic light‐emitting diodes based on these emitters, diluted in oligostyrene, exhibit external quantum efficiencies of around 12%.

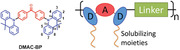

## 1 Introduction

Since the first report of organic light emitting diodes (OLEDs) based on emitters that exhibit thermally activated delayed fluorescence (TADF),^[^ ^1^ ^]^ effort has focused on understanding the underlying physical processes,^[^ ^2^, ^3^ ^]^ as well as improving performance by optimizing chemical design^[^ ^4^, ^5^, ^6^, ^7^ ^]^ and device architecture.^[^ ^8^, ^9^, ^10^ ^]^ In contrast to conventional luminescent materials, TADF emitters exhibit a small energy difference (Δ*E_ST_*) between the lowest singlet (S1) and triplet (T1) states, promoting an efficient reverse intersystem crossing (RISC). This process enables harvesting triplet excitons, which are non‐emissive, except in case of heavy metal‐based emitters. Since the latter are non‐desirable from an environmental point‐of‐view, TADF materials are attractive alternatives. A low Δ*E_ST_* is achieved by combining an electron donating (D) and an electron accepting (A) moiety into a single chromophore in a near orthogonal arrangement, due to which the spatial overlap between the highest occupied and lowest unoccupied molecular orbitals (HOMO and LUMO) is small.^[^ ^11^ ^]^ This D–A arrangement introduces a charge transfer (CT) state exhibiting a significant electronic coupling with the local triplet state in order to facilitate efficient TADF.^[^ ^12^ ^]^

In recent years, highly efficient OLEDs based on small molecule TADF emitters (SM‐TADF) have been developed.^[^ ^13^ ^]^ Although major advances in quantum efficiencies, lifetimes, and full‐color range emitters have been achieved, vacuum deposition, which is the commonly applied deposition method for SM‐emitters, limits scalability both from a practical and cost perspective.^[^ ^14^ ^]^ In contrast, soluble TADF polymers allow for both cost‐effective and scalable manufacture through coating and printing. In a typical OLED architecture, the TADF based emitter is dispersed in a wide band‐gap host.^[^ ^15^, ^16^, ^17^ ^]^ Application of such a host material has two major advantages with regard to the OLED efficiency: first, it avoids self‐quenching between the emitters. Second, the negative effects of charge carrier trapping, such as unbalanced transport and non‐radiative recombination via trap states, are strongly reduced by the trap‐dilution effect.^[^ ^18^ ^]^

Most commonly, such a host is a co‐evaporated small molecule with energy levels outside the bandgap of a small molecule TADF (SM‐TADF) emitter. As thermal evaporation of such systems occurs in a kinetically controlled regime, the emitter and the host are usually well mixed in the resulting film. In contrast, in case of solution processing, molecular mobilities remain significant during film forming due to the presence of a solvent. This may result in non‐desirable mass transport and phase separation. There are hence two additional criteria in case of solution processed systems: i) miscibility between the solutes and between the solutes and the solvent; and ii) good film‐forming properties through a strongly concentration‐dependent viscosity. With respect to the latter, the use of polymeric emitters is advantageous over small molecular ones, whereby we note that in case of a blend the host is preferably polymeric as well. Another advantage of using polymeric systems is that the charge transport properties are expected to be less affected by diluting them in a large band gap host due to the improved percolation as compared to small molecules. A caveat, however, is that polymers are notoriously difficult to mix due to the (strongly) reduced translational entropy.^[^ ^19^ ^]^ Hence, efficient polymeric emitters hinge on smart molecular design, not only to achieve optimal electronic properties, but also good miscibility and processability.

State‐of‐the‐art TADF polymers can be categorized according to design.^[^ ^14^ ^]^ A first category represents polymers where both D and A are embedded in the backbone. In this class, D–A alternating and fully conjugated^[^ ^15^, ^17^ ^]^ as well as insulating linker‐separated main‐chain polymers^[^ ^15^, ^16^, ^20^ ^]^ have been reported. Other architectures are backbone‐D/pendent‐A polymers,^[^ ^21^, ^22^, ^23^, ^24^ ^]^ as well as polymers with a non‐conjugated backbone bearing the D–A TADF chromophores grafted as pendant moieties. Whereas conjugated linkers generally provide for better charge transport than non‐conjugated ones, the increased conjugation length may result in the energy of the locally excited triplet state to decrease below that of the charge‐transfer triplet state. For this reason, the degree of conjugation is often tempered by using a wide bandgap aromatic system and/or introducing steric or topological constraints.^[^ ^15^, ^25^ ^]^ Miscibility, solubility, and processability also depend on polymer design, since they are not only affected by backbone flexibility and side groups, but also on linker chemistry. Unfortunately, systematic studies consistently comparing different linker moieties towards an optimal TADF polymer design are sparse.

In this work, we present a range of novel main‐chain TADF polymers wherein the bis[4‐(9,9‐dimethyl‐9,10‐dihydroacridine)phenyl]methanone (DMAC‐BP) D–A–D chromophore^[^ ^17^ ^]^ is coupled via a set of consistently varied linker units. The set includes rigid, conjugated (phenyl), as well as flexible, non‐conjugated (alkyl) spacers, which are varied in length. After outlining their synthesis in detail, we characterize the polymers in reference to the monomeric DMAC‐BP derivative using steady state and time‐resolved spectroscopy, both in solution and in the solid film. The measurements on the pristine films demonstrate that after polymerization the TADF properties are retained. This is reflected by the equivalent performance of the small molecule and polymer blend films in OLEDs, exhibiting a >10% external quantum efficiency (EQE). Finally, we identify another marked advantage of using polymers over SM‐emitters, as devices based on the latter exhibit a marked decrease in EQE at similar dilution, likely due to a loss of charge percolation.

## 2 Results and Discussion

**Figure 1:**
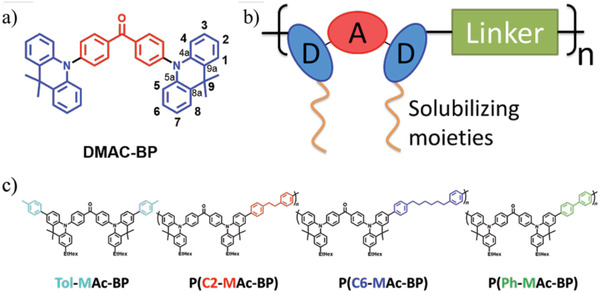
a) Molecular structure of the DMAC‐BP TADF chromophore, used as the basis for this work. b) Schematic representation of the general design of our DMAC‐BP‐based TADF polymers. c) Molecular structures of the DMAC‐BP‐based TADF polymers, as well as the monomeric reference chromophore (Tol‐MAc‐BP).

Bis[4‐(9,9‐dimethyl‐9,10‐dihydroacridine)phenyl]methanone (DMAC‐BP) was selected as TADF model system. This chromophore combines a benzophenone unit as acceptor with a 9,9‐dimethyl‐9,10‐dihydroacridine moiety as donor (see **Figure** **1**a). Our choice to use DMAC‐BP was motivated by the extremely small Δ*E_ST_* of 7 meV and a high PLQY of 0.85 in neat film, showing only a small concentration quenching (0.90 for 10 wt% diluted in m‐bis(N‐carbazolyl)benzene [mCP] film).^[^ ^26^ ^]^ The authors ascribe the limitation of concentration quenching to the inhibited interaction between the donors due to the methyl substitution of the DMAC groups. We designed a range of DMAC‐BP based polymers through connecting the acridine donors at the 2‐position with a linker moiety (Figure 1b). This way, the D–A–D triad is accommodated in the polymer backbone, leading to an identical substitution pattern on the donor units. Furthermore, 2‐ethylhexyl (EtHex) side chains were introduced as solubilizing moieties at the 7‐position of the acridine moieties to ensure solubility of the polymers. The set of materials (Figure 1c) consists of a fully conjugated polymer P(Ph‐MAc‐BP), based on a 1,1′ biphenyl linker, as well as two non‐conjugated polymers P(C2‐MAc‐BP) and P(C6‐MAc‐BP), respectively based on 1,2‐diphenylethane and 1,6 diphenylhexane as short/stiff and long/flexible linker moieties. To suitably compare the properties of the TADF polymers to that of a structurally similar monomeric chromophore, we chose a modified version of DMAC‐BP, bearing phenyl and ethylhexyl groups at the 2‐ and 7‐positions of the acridine (Tol‐MAc‐BP).

**Figure 2:**
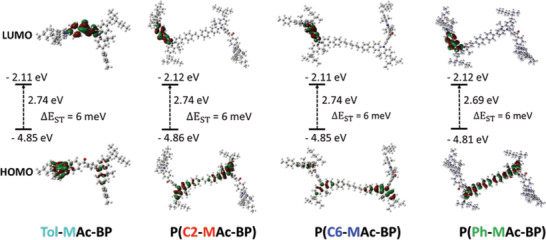
Frontier orbital distributions of the TADF emitters synthesized in this work, calculated using DFT.

Before detailing the synthesis of the TADF emitters, we present their electronic properties as predicted by density functional theory (DFT) calculations on oligomers comprising two D–A–D triads, connected by the respective the linkers. The ground‐state geometries were optimized at B3LYP/6‐31G(d,p) level and in subsequent time‐dependent DFT (TD‐DFT) calculations the energies of the S_1_ and T_1_ levels were determined. The results are displayed and summarized by **Figure** **2** and **Table** **1**. These vacuum calculations demonstrate that irrespective of the type of linker, upon polymerization the HOMO and LUMO remain strongly localized on, respectively, the acridine and benzophenone units, without exhibiting loss in orthogonality. In other words, the dihedral angle between the donor and acceptor close is close to 90° in all cases. The only clear deviation from an electronic point of view is that the HOMO of the fully conjugated polymer P(Ph‐MAc‐BP) extends from the acridine units across the linker. Nevertheless, the HOMO and LUMO energies of the polymers are comparable to those of the monomeric reference and in all cases Δ*E_ST_* remains very low and unaffected by the presence of the linker unit. Hence, the electronic prerequisites for TADF are retained upon polymerization of the DMAC‐BP chromophores. This is an important result, as it excludes the attribution to profound differences in intrinsic molecular properties of any observed dependence of the optoelectronic behavior on the linker chemistry.

**Table 1:** Results from DFT calculations

Emitter	*E_HOMO_* [eV]	*E_LUMO_* [eV]	Δ*E*_*HOMO* − *LUMO*_ [eV]	$E_{S_{1}}$ [eV]	*E_T_* [eV]	Δ*E_ST_* [meV]	Θ_*D* − *A*_ ^a)^ [°]	Θ_*D*_ ^b)^ [°]
Tol‐MAc‐BP	4.847	2.109	2.739	2.241	2.235	6	89.2	179.3
P(C2‐MAc‐BP)	4.861	2.116	2.746	2.254	2.248	6	89.1	179.3
P(C6‐MAc‐BP)	4.853	2.112	2.741	2.245	2.239	6	89.3	178.8
P(Ph‐MAc‐BP)	4.805	2.120	2.685	2.235	2.229	6	88.2	179.6

^a)^ Average of dihedral angles between donor and acceptor in the D–A–D triad;

^b)^ Average of dihedral angles along N—C^4a^ and N—C^5a^ bond in D.

**Scheme 1:**
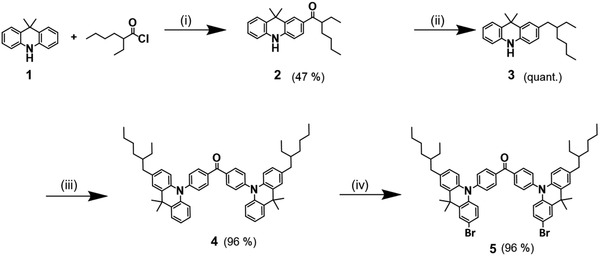
Synthesis of the TADF comonomer 5: i) AlCl_3_, CH_2_Cl_2_, −20 °C → r.t.; ii) LiAlH_4_, AlCl_3_, THF, 0 °C → r.t; iii) 4,4′‐dibromobenzophenone, Pd(t‐Bu_3_P)_2_, K_2_CO_3_, toluene, 100 °C; and iv) *N*‐bromosuccinimide, THF, r.t.

In what follows, we detail the synthesis of all four DMAC‐BP‐based TADF polymers, as well as of the monomeric chromophore Tol‐MAc‐BP. Each polymer comprises two comonomer precursors, which react in a Suzuki polycondensation: i) the dihalogenated D–A–D chromophore; and ii) the linker units, end‐functionalized with boronic acid pinacol ester groups. The synthesis of the D–A–D chromophore^[^ ^17^ ^]^ is depicted in **Scheme** **1**. The commercially 9,10‐dihydro‐9,9‐dimethylacridine (1) was substituted with 2‐ethylhexanoyl chloride via a Friedel–Crafts acylation to obtain the asymmetric ketone 2. After a reduction towards mono(ethylhexylacridine) 3, a Buchwald–Hartwig amination with 4,4‐dibromobenzophenone was carried out to produce the donor‐acceptor‐donor chromophore 4 in near quantitative yield. The latter was subsequently dibrominated to yield the final comonomer 5 in an overall yield of 43%.

**Scheme 2:**
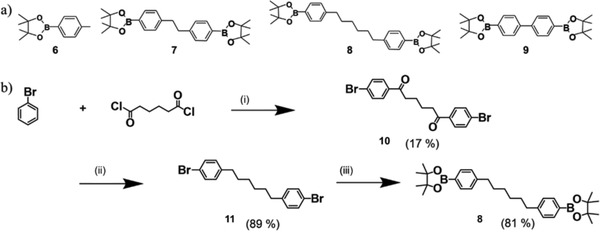
a) Di‐boronic acid pinacol ester precursors of the linker comonomers. b) Synthesis of linker comonomer precursor 8: i) AlCl_3_, 50 °C; ii) trifluoroacetate (TFA), triethylsilane, CH_2_Cl_2_, r.t.; iii) 1) n‐butyl lithium, THF, −78 °C and 2) 2‐isopropoxy‐4,4,5,5‐tetramethyl‐1,3,2‐dioxaborolane, −78 °C → r.t.

Of the boronic‐ester functionalized 1,1′‐biphenyl linker comonomer precursors 6–9 (see **Scheme** **2**a), only the 1,6‐diphenylhexane derivative 8 is not commercially available. Its synthesis^[^ ^27^, ^28^ ^]^ is therefore depicted in Scheme 2b. Starting from bromobenzene and adipoyl dichloride, intermediate 10 was formed in a double Friedel–Crafts acylation para to the bromide residues. In a second step, the ketones were reduced to yield dibromide 11 in high yield. In a final step, the bromides were substituted by a boronic acid pinacol ester, affording linker comonomer precursor 8 in an overall yield of 12%.

**Scheme 3:**
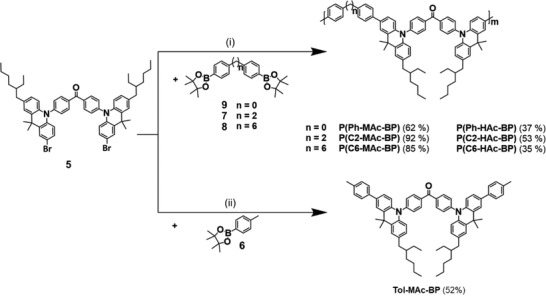
Syntheses of TADF polymers P(C2‐MAc‐BP), P(C6‐MAc‐BP), and P(Ph‐MAc‐BP), as well as the monomeric TADF chromophore Tol‐MAc‐BP: i) 1) Pd(PPh_3_)_4_, Na_2_CO_3_, toluene/EtOH/H_2_O, 120 °C and 2) 6, Pd(PPh_3_)_4_, 120 °C; ii) Pd(PPh_3_)_4_, Na_2_CO_3_, toluene/EtOH/H_2_O, 120 °C.

The set of TADF materials was synthesized in a final Suzuki–Miyaura (poly)condensation between boronic acid pinacol esters 6–9 and the halogenated TADF precursor 5 (see **Scheme** **3**). This reaction was performed in a sealed pressure tube for 72 h with a biphasic mixture of toluene, water, and ethanol. The small molecule Tol‐MAc‐BP was obtained in a 52% yield. The polymers were end‐capped after the 72 h by adding 9, as well as fresh catalyst and allowed to react for an additional another 24 h. The motivation for the end capping is the removal of any halogen residues, that otherwise would possibly have led to enhanced trapping and excited state quenching. Upon purification via Soxhlet extraction and preparative size exclusion chromatography (SEC), the DMAC‐BP‐based polymers were obtained in yields of 62–92%. Their full characterization, as well as that of the monomeric reference chromophore Tol‐MAc‐BP is given in Section S1, Supporting Information. The molecular weight distributions of the polymeric emitters were determined using GPC (see Figure S2 and Table S1, Supporting Information). The polymers exhibit similar molecular weight distributions with a number average molecular weight (${\bar{M}}_{n}$) ≈6 kg mol^−1^ and dispersities (*Đ*) ≈1.9.

A prerequisite for a long OLED life time is a high thermal stability^[^ ^29^ ^]^ (i.e., no chemical degradation), as well as a high morphological stability (i.e., no phase transitions and/or thermal transitions). To establish this, we subjected the TADF chromophores to thermogravimetric analysis (TGA) and differential scanning calorimetry (DSC). All materials show a high thermal stability with a decomposition temperature (i.e., at 5% weight‐loss) in the range 425–435 °C. The absence of crystallization and melting peaks in the DSC demonstrates that the materials are fully amorphous. The glass transition temperatures of the polymers are well in excess of 100 °C. In other words, from a thermal stability perspective, the TADF polymers are very suitable for application in OLEDs.

**Figure 3:**
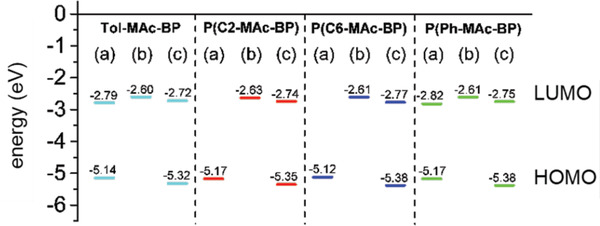
Estimates for the HOMO and LUMO energies of the TADF chromophores. a) Approximated by, respectively, the ionization potential (*IP_CV_*) and electron affinity (*EA_CV_*) directly obtained from CV. b) LUMO energy approximated by adding the optical gap (around *E*_g_ = 2.63 eV for all materials) to *IP_CV_* c) HOMO approximated by the ionization potential obtained from UPS (*IP_UPS_*) and the LUMO by adding the optical gap to *IP_UPS_*.

To enable the selection of suitable materials for the hole‐ and electron‐injection layers for OLEDs based on the novel TADF emitters, we determined their HOMO and LUMO levels using a combination of (solution) UV–vis spectroscopy, cyclic voltammetry (CV), and (solid state) ultraviolet photoelectron spectroscopy (UPS) (see Section S2, Supporting Information, for details). Since the estimates from different methods may vary, we applied different ways to approximate the energy levels based on the values for the ionization potential (*IP*) measured by both CV and UPS, the electron affinity (*EA*), obtained from CV, and the optical band gap (*E_g_*), obtained from UV–vis. Details of these measurements are in Section S1, Supporting Information. **Figure** **3** shows that the HOMO and LUMO energies are similar for all emitters, demonstrating that the linker type has no major influence on the intrinsic electronic properties, in principle agreement with the DFT calculations.

In order to demonstrate to what extent the TADF properties of the DMAC‐BP unit are retained after polymerization, we analyzed solid films of the materials using time‐resolved photoluminescence (TRPL) spectroscopy, by measuring transient PL decay curves. To minimize self‐quenching, we blended the materials with PS as a large bandgap host in a 1:9 TADF‐chromophore:PS weight ratio. For this, low molecular weight PS (${\bar{M}}_{n}$ = 1.2 kg mol^–1^) was used, since a high molecular weight PS (${\bar{M}}_{n}$ = 170 kg mol^–1^) resulted in phase separation. This molecular weight dependence is explained by Flory–Huggins theory, which states that the entropy of mixing, which favors mixing, scales inversely with chain length.^[^ ^19^ ^]^ In a previous work, we have demonstrated this by comparing calculated phase diagrams for binary solutions of the conducting polymer poly(diethylhexyl‐*p*‐phenylenevinylene) (BEH‐PPV) and PS at various molecular weights with AFM images of the thin film morphologies.^[^ ^30^ ^]^ Indeed, a molecular weight of, in this case, ${\bar{M}}_{n}\left(\mathrm{PS}\right)$ = ≈35 kg mol^–1^ led to extensive phase separation, in agreement with a large miscibility gap in the calculated phase diagram. In contrast, for ${\bar{M}}_{n}\left(\mathrm{PS}\right)$ = ≈1 kg mol^–1^ no phase separation was observed even despite the high molecular weight of the BEH‐PPV, together with a near absence of a coexistence region. In Section S6, Supporting Information we give exemplary AFM images of blend films (Figure S13, Supporting Information), which show that for low molecular weight PS as a host, our TADF materials do not phase separate.

**Figure 4:**
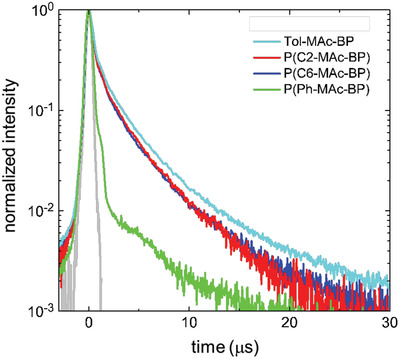
Normalized transient PL decay curves of 10 wt% films of the TADF polymers and small molecular reference Tol‐MAc‐BP in PS as a host (*λ*_exc_ = 400 nm, *λ*_em_ = 520–540 nm). *T* = 295 K.

**Figure** **4**, which plots the TRPL traces measured at a temperature of *T* = 295 K, shows that for all materials the fluorescence intensity exhibits a decay over several tens of microseconds, owing to the presence of a delayed component. The emission lifetime of the non‐conjugated polymers P(C2‐MAc‐BP) and P(C6 MAc‐BP) is very similar to that of the monomeric Tol‐MAc‐BP, whereas that of the fully conjugated P(Ph‐MAc‐BP) is considerably shorter. No significant difference based on the length of the alkyl linker is observed between the decay curves of P(C2‐MAc‐BP) and P(C6‐MAc‐BP), which shows that intrachain quenching between neighboring TADF units is not influenced by the length of the alkyl linker.

**Figure 5:**
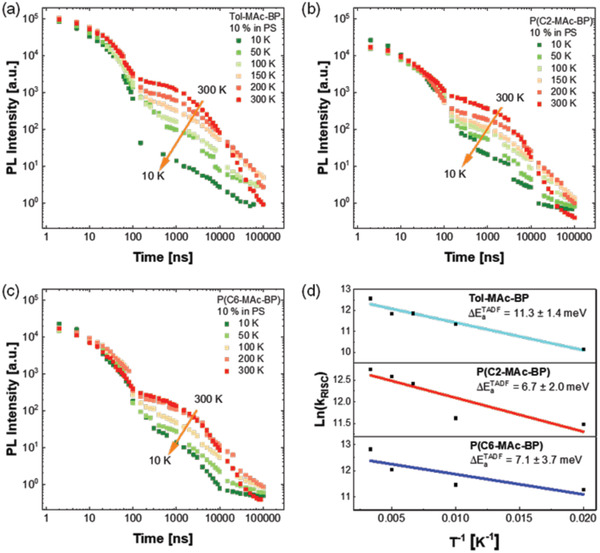
Transient PL decay curves of films of films of a) Tol‐MAc‐BP, b) P(C2‐MAc‐BP), and c) P(C6‐MAc‐BP), 10 wt% in PS, recorded at different temperatures in helium. d) Arrhenius plot of the RISC rate of Tol‐MAc‐BP, P(C2‐MAc‐BP), and P(C6‐MAc‐BP) derived from (a–c) and fitted with a straight line.

As next step, we measured the luminescence decay as a function of excitation power and temperature to establish whether the delayed fluorescence (DF) is indeed due to RISC and not originating from triplet–triplet annihilation (TTA). Since the latter is a bimolecular process, DF due to TTA would exhibit a quadratic dependence on low laser power.^[^ ^31^, ^32^ ^]^ In contrast, TADF is a monomolecular process, resulting in a linear dependence. In all cases, a linear proportionality between the integrated DF and the excitation power was found (see Figure S6, Supporting Information), demonstrating TADF to be responsible for the DF. The temperature‐dependent TRPL traces are given in **Figure** **5**. In these measurements we have omitted the conjugated P(Ph‐MAc‐BP) due to its strongly suppressed emissive properties. The TRPL kinetic traces shown in Figure 5 exhibit two decay regimes. The faster decay, between 1–10^2^ ns, is due to prompt fluorescence from S_1_ populated by photoexcitation. The slower decay ranges from 10^2^–10^5^ ns and is attributed to DF, arising from repopulation of the S_1_ state from T_1_. In all cases the DF intensity decreases with temperature, demonstrating thermally activated behavior. The DF remains apparent at 10 K, consistent with the small Δ*E_ST_*. The TADF activation energy, obtained from an Arrhenius analysis of the rate constant for RISC,^[^ ^33^, ^34^ ^]^ ranges from 6.7 to 11.3 meV (Figure 5d), in good agreement with the DFT calculations. The value is not influenced by the length of the alkyl linker. Subtracting these values from the estimates for the optical gap (see Section S3, Supporting Information), gives an estimate for the triplet level of T_1_ = 2.59, 2.60, and 2.64 eV for Tol‐MAc‐BP, P(C2‐Mac‐BP), and P(C6‐MAc‐BP), respectively. We note however, that, in view of the experimental error and the extremely small Δ*E_ST_*, the differences likely do not have a physical significance, but are all in good agreement with the values reported for DMAC‐BP.^[^ ^26^ ^]^

Next, we investigate the charge transport in our TADF emitters. For this, we fabricated single‐carrier devices based on all four (pristine) materials. We refer to the Experimental Section for details regarding the device structure. For the hole‐only (HO) devices, we used p‐pTFF‐C_2_F_5_SIS (full name in the Experimental Section) as a hole‐injection layer.^[^ ^35^ ^]^ This self‐doped polymer has a high work function of 5.85 eV and therefore aligns well with the relatively deep HOMO levels of our TADF materials. On top of the TADF layer we use the high‐work function material molybdenum oxide (MoO_3_),^[^ ^36^ ^]^ to prevent electron injection. In the electron‐only (EO) devices, the emitter is situated between two low work function electrodes, for which we used Al(30 nm) and Ba(5 nm)/Al(100 nm) as bottom and top electrode, respectively (see Experimental Section).

**Figure 6:**
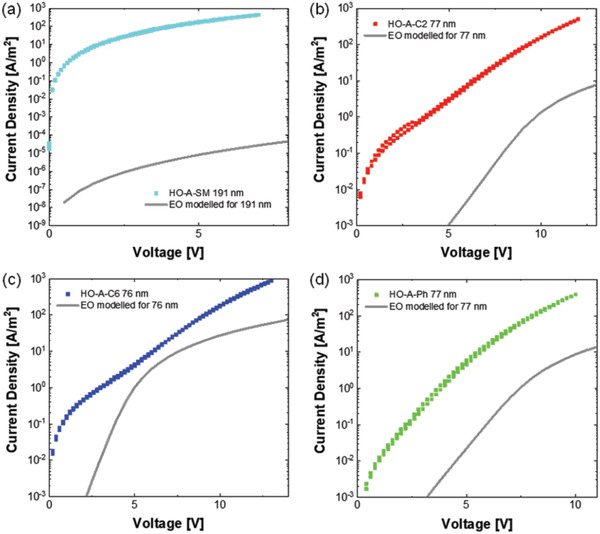
Current density plotted as a function of voltage of single carrier devices based on a) Tol‐MAc‐BP, b) P(C2‐MAc‐BP), c) P(C6‐MAc‐BP), and d) P(Ph‐MAc‐BP). The squares represent measured hole current densities measured at 295 K and the lines represent simulated electron current densities, assuming the same layer thickness as in the corresponding hole‐only device (see legend) and taking input from measured and fitted EO data (Section S4, Supporting Information).

The hole‐ and electron‐current densities at *T* = 295 K are plotted as a function of voltage in **Figure** **6**. For the full set of the single carrier *J* − *V* characteristics, we refer to Figures S7–S9, Supporting Information. To directly compare the hole and electron currents, we corrected for the film thickness of the electron‐only devices by using numerical drift‐diffusion simulations,^[^ ^37^, ^38^ ^]^ which were first fitted to the experimental electron currents. Despite the fact that in the hole‐only devices the film thickness of the polymers is more than a factor two lower, the small molecule Tol‐MAc‐BP (see Experimental Section) exhibits the highest hole current. This indicates that polymerization leads to a reduction in the hole conduction properties of the TADF‐emitter. Furthermore, the relatively steep voltage dependence indicates the presence of hole traps. For P(C2‐MAc‐BP) we obtained a hole mobility of 4 × 10^–10^ m^2^ V^–1^ s^–1^, as well as a hole trap density of 7.2 × 10^23^ m^–3^. For the width of the Gaussian density of states DOS, we obtained 0.08 eV.^[^ ^39^ ^]^

Figure 6 shows that the electron current density is generally lower than for the holes and also exhibiting a relatively steep voltage dependence. Curve fitting (Section S4, Supporting Information) gave an electron mobility ranging from 4 × 10^–12^ m^2^ V^–1^s^–1^ for P(C2‐MAc‐BP) to 3 × 10^–11^ m^2^ V^–1^s^–1^ for P(C6‐MAc‐BP). Interestingly, the electron mobility of the fully conjugated P(Ph‐MAc‐BP) is of the same magnitude as for the polymers based on non‐conjugated linkers. For all materials, the electron current is lower than the hole current, despite the fact that the electron trap density was found to be of similar magnitude, that is, ≈10^23^ m^3^. A similar unbalance in the charge carrier transport is seen for many fluorescent organic emitters^[^ ^18^, ^40^, ^41^ ^]^ and can be counteracted in the OLED by applying an electron transport layer, which also acts as a hole‐blocking material. Interestingly, Figure 6 shows that the charge transport in the polymers is actually more balanced than in the small molecular emitter.

As a final step, we fabricated OLEDs based on our TADF materials as the emissive layer (EML). We refer to the Experimental Section for details on the fabrication procedure and device structure. We used a simple bilayer architecture with a layer of the emitter and 2,2″,2‴‐(1,3,5‐benzinetriyl)‐tris(1‐phenyl‐1‐H‐benzimidazole) (TPBi) as an electron transport/hole blocking layer. Furthermore, the TADF materials were diluted in PS with blending ratios of 1:0 (pristine emitter), 1:1, 1:3, and 1:9. The electroluminescence (EL) spectra of all materials, plotted as a function of degree of dilution in polystyrene in Figure S12, Supporting Information, exhibit a single band ≈540 nm, comparable to the photoluminescence spectra. A small blueshift of the band is observed with increasing polystyrene content, which we ascribe to the solvatochromism associated with the charge‐transfer nature of the excited state of TADF chromophores.^[^ ^3^ ^]^

**Figure 7:**
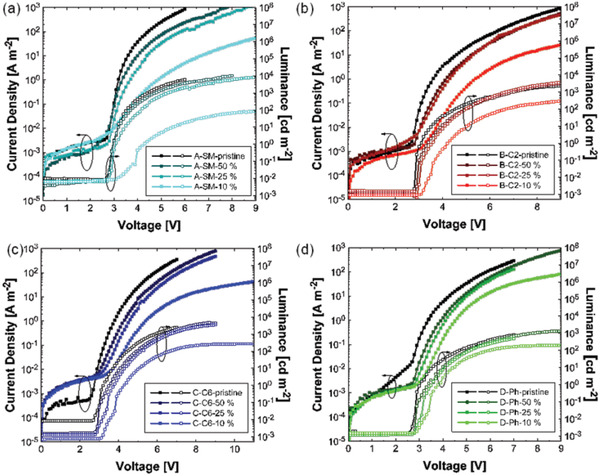
*J* − *V* − *L* characteristics of devices with a) Tol‐MAc‐BP, b) P(C2‐MAc‐BP), c) P(C6‐MAc‐BP), and d) P(Ph‐MAc‐BP) as emitter, blended with PS in various ratios as indicated in the legends.

The current–voltage–luminescence (*J* − *V* − *L*) characteristics are given in **Figure** **7**. An interesting observation is that upon dilution of the TADF small molecule Tol‐MAc‐BP in PS the current density drops significantly in comparison to the pristine film. In contrast, the TADF polymers show a smaller relative decrease in current density upon dilution. This trend can be explained by the notion that since Tol‐MAc‐BP is a small molecule, percolating pathways for charge carriers are more easily lost upon diluting than for the polymeric emitters. Therefore, the polymer emitters can retain their charge transport upon dilution, which positively affects the EQE.

**Figure 8:**
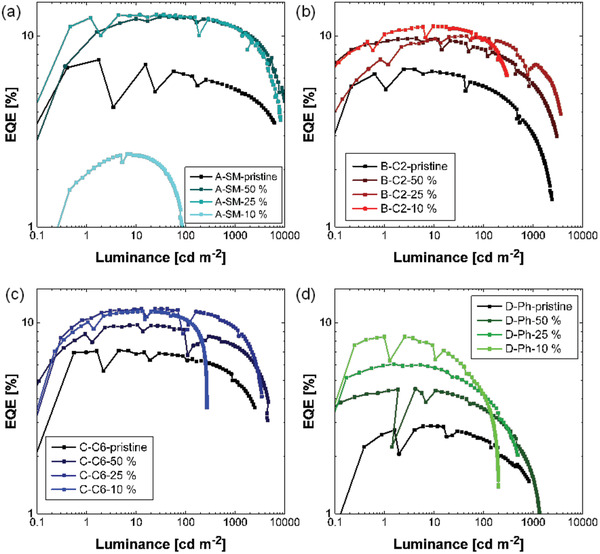
EQE, plotted as a function of luminance of OLED devices based on a) Tol‐MAc‐BP, b) P(C2‐MAc‐BP), c) P(C6‐MAc‐BP), and d) P(Ph‐MAc‐BP) as emitting materials, blended with PS in various ratios as indicated. The “jumps” in the value for the EQE is a measurement artifact visible at low photocurrents, likely related to the analyzer internally changing scale.

The EQE of the devices based on unblended Tol‐MAc‐BP, P(C2‐MAc‐BP), and P(C6‐MAc‐BP) already surpass 5%, clearly demonstrating the occurrence of TADF (see **Figure** **8**), since the EQE of an OLED based on a conventional fluorescent emitter reaches a maximum of about 5%, due to a ≈20% light outcoupling efficiency, and 25% singlet‐exciton emission. Only for the devices based on the conjugated P(Ph‐MAc‐BP) the EQE is substantially lower, which can be attributed to less efficient TADF, evidenced by the less intense delayed emission in the TRPL trace (Figure 4). Upon diluting the emitters in PS, the EQEs are boosted to values well above 10%, owing to reduced self‐quenching. Dilution even increased the EQE of the P(Ph‐MAc‐BP) device beyond 5%. We note that for these devices no special measures were taken to increase light outcoupling and using only simple device structure with a solution‐processed emissive layer in combination with an electron‐transport layer.

As charge trapping was observed in all emitters, losses due to non‐radiative trap‐assisted recombination could be present.^[^ ^40^ ^]^ Interestingly, the performance of the Tol‐MAc‐BP‐based devices decreases significantly at high dilution, compared to those based on the diluted non‐conjugated polymeric emitters, again likely due to the loss of charge percolation. This demonstrates the advantage of polymerized TADF emitters, where reduced concentration quenching does not go at the expense of charge transport. To confirm that the device performance is not suppressed by the fact that polystyrene is an insulating host, possibly negatively affecting charge transport, we produced an identical set of devices but now based on tris(4‐carbazoyl‐9‐ylphenyl)amine (TCTA) and 1,1‐bis[(di‐4‐tolylamino)phenyl]cyclohexane (TAPC) as conducting host materials (see Section S4, Supporting Information).^[^ ^17^, ^42^ ^]^ Indeed, Figure S11, Supporting Information, shows that the *J* − *V* − *L* characteristics, as well as the EQEs of these devices are indeed comparable to the ones based on the emitting layer comprising the polystyrene host.

## 3 Conclusions

High performance OLEDs were achieved, based on novel green‐emitting TADF polymers as highly efficient organic emitters. These polymers contain DMAC‐BP‐type donor–acceptor–donor chromophores, coupled via various conjugated and non‐conjugated (alkyl) linkers. Upon diluting the TADF polymers in low molecular weight polystyrene as a host, the fluorescence of, in particular, the ones with non‐conjugated C_2_ and C_6_ alkyl linkers exhibited a significant delayed component owing to TADF. The current–voltage characteristics of single charge carrier devices of the pristine polymers showed that in comparison to the monomeric reference emitter, charge transport is on the one hand not unaffected by traps, but on the other rather balanced. OLED devices with emissive layers based on the non‐conjugated TADF polymers diluted in polystyrene, exhibited EQE well over 10%, in the absence of structures and/or layers that enhance light outcoupling. The EQE of the polymer‐based devices furthermore proved robust against high dilution down to 10% of emitter, which is attractive from a materials cost point‐of‐view. In contrast, dilution of the monomeric emitter resulted in a significant loss in EQE, likely due to the interruption of charge percolation. Our results show that the use of alkyl linkers is a viable strategy in developing highly efficient OLEDs based on solution‐processable main‐chain TADF polymers.

## 4 Experimental Section

### Materials and Materials Analysis

All chemical precursors and reactants were acquired from standard suppliers and used as received. The synthesis of precursors 2, 3, 4, 5, 8, 10, and 11 has been carried out according to literature procedures.^[^ ^17^, ^27^, ^28^ ^]^ DSC was performed on Thermal Analysis DSC 3+ from Mettler Toledo. Thermogravimetric analysis (TGA) was performed on a TGA/DSC 3+ – Thermogravimetric Analyzer with high temperature furnace (HT) from Mettler Toledo under nitrogen flow with a heating rate of 10 °C min^−1^ from 25 to 300 °C.

### Cyclic Voltammetry

Cyclic voltammetry (CV) measurements were carried out on a Metrohm Autolab PGSTAT204 potentiostat/galvanostat with a three‐electrode‐cell system: glassy carbon electrode as the working electrode, Ag/AgCl electrode as the reference electrode, platinum wire as the counter electrode, and Bu_4_NPF_6_ (0.1 m in dichloromethane) as supporting electrolyte with a scan rate of 100 mV s^−1^. Before recording the CV the solution was sparked with argon for 3 min and an argon stream was kept flushing the headspace during the measurement. Ferrocene was used as an internal reference.

### Ultraviolet Photoelectron Spectroscopy (UPS)

See Section S1, Supporting Information.

### Steady State Spectroscopy

Ultraviolet–visible (UV–vis) absorption spectra were recorded on a Perkin Elmer Lambda 900 UV/Vis/NIR spectrometer using the software Perkin Elmer UV Winlab (version 6.04.0738). Steady state photoluminescence spectra were recorded with a HORIBA Jobin‐Yvon Fluorolog 3–22 Tau‐3 using a photomultiplier tube (PMT) as detector.

### Time‐Resolved Spectroscopy

Solid films for time resolved spectroscopy analysis were prepared by mixing the TADF chromophores with atactic polystyrene (${\bar{M}}_{n}$ = 1.2 kg mol^−1^, *Đ* = 1.02) in a 1:9 w/w ratio at a total solids concentration of 20 mg mL^−1^ in chlorobenzene, followed by spin coating and drying. Time‐resolved photoluminescence (TRPL) measurements were carried out on two different setups. In both cases, the samples were photoexcited at 400 nm using the frequency‐doubled output from a Ti:Sapphire laser (Coherent, Libra HE) supplying 100 fs pulses with a repetition rate of 1 kHz. The initial characterization of the fluorescence lifetimes at *T* = 295 K were measured using a streak camera (Hamamatsu C5680, slow sweep mode; 50 µs time window: 0.23 µs instrument response) and HPDTA (version 9.5pf7) was used as operating software. The PL decay was collected at the PL maximum of the delayed fluorescence (520–540 nm). In order to limit triplet excited state quenching by oxygen, the sample was measured in an encapsulated sample holder in nitrogen atmosphere and loaded in a glovebox. The power and temperature dependent TRPL measurements were recorded using a 4‐Picos gated iCCD camera (Stanford Computer Optics). 4spec (version 2.30.0.2) was used as operating software. The film samples were loaded into a top load exchange gas closed cycle cryostat (GMX‐19‐OmniPlex, Advanced Research Systems, Inc.). To ensure a helium atmosphere, the cryostat was first flushed with dry nitrogen for 2 min and then with helium for 30 s. The measurements were performed under this helium atmosphere for the entire temperature range.

### Single Carrier Devices

To fabricate hole‐only (HO) devices, a 40 nm layer of p‐pTFF‐C_2_F_5_SIS^[^ ^43^ ^]^ (see molecular structure in Figure S3, Supporting Information) was applied onto glass/ITO (100 nm) via spin coating from acetonitrile. Subsequently, the emitter was spin coated from chlorobenzene, after which a 10 nm layer of molybdenum oxide (MoO_3_) was thermally evaporated and covered with a 100 nm evaporated aluminum top electrode to give the following device structure: glass/ITO (100 nm)/p‐pTFF‐C_2_F_5_SIS (40 nm)/TADF emitter/MoO_3_ (10 nm)/Al (100 nm). The TADF‐polymers give a film thickness of ≈76 nm, whereas Tol‐MAc‐BP gave 191 nm. Electron‐only (EO) were prepared by evaporating 30 nm of aluminum on glass, followed by spin coating of the TADF emitter and thermal evaporation of, respectively, 5 nm barium and 100 nm aluminum as top electrode. This gave the following device structure: glass/Al (30 nm)/TADF emitter/Ba (5 nm)/Al (100 nm).

### Organic Light‐Emitting Diodes

OLEDs with the following device structure: ITO (100 nm)/p‐pTFF‐C_2_F_5_SIS (40 nm)/EML (30 nm)/TPBi (60 nm)/Ba (5 nm)/Al (100 nm), were prepared by spin coating p‐pTFF‐C_2_F_5_SIS from acetonitrile onto ITO‐covered glass substrates, followed by spin coating the EML (blend of emitter and PS) from chlorobenzene. The TPBi layer and subsequently the BaAl cathode were applied by thermal evaporation.

## Conflict of Interest

The authors declare no conflict of interest.

## Supporting information

Supporting Information

## Data Availability

The data that support the findings of this study are available from the corresponding author upon reasonable request.
