# Supplementary material for: Role of Linker Functionality in Polymers Exhibiting Main‐Chain Thermally Activated Delayed Fluorescence
Source: Adv Sci (Weinh). 2022 Mar 6;9(19):2200056. doi: 10.1002/advs.202200056 (PMC9259719; doi:10.1002/advs.202200056)
Supplement: Supplementary file 1 — Supporting Information [file ADVS-9-2200056-s001.pdf]

ADVANCED SCIENCE
Open Access

Supporting Information

for *Adv. Sci.*, DOI 10.1002/advs.202200056

Role of Linker Functionality in Polymers Exhibiting Main-Chain Thermally Activated Delayed Fluorescence

*Kai Philipps, Yutaka Ie, Bas van der Zee, Rui-Qi Png, Peter K. H. Ho, Lay-Lay Chua, Esther del Pino Rosendo, Charusheela Ramanan, Gert-Jan A. H. Wetzelaer, Paul W. M. Blom and Jasper J. Michels**

Supporting Information

Role of Linker Functionality in Polymers exhibiting Main-Chain Thermally Activated Delayed Fluorescence

*Kai Philipps, Yutaka Ie, Bas van der Zee, Rui-Qi Png, Peter K. H. Ho, Lay-Lay Chua, Charusheela Ramanan, Gert-Jan Wetzelaer, Paul W.M. Blom and Jasper J. Michels**

S1. Synthesis and materials characterization

Synthesis of ***Tol-MAc-BP***

A modified procedure based on literature was carried out.[1] 4,4,5,5-Tetramethyl-2-(p-tolyl)-2,1,3,2-dioxaborolane (**6**; 130 mg, 0.59 mmol, 3.5 eq.), Na_2CO_3 (358 mg, 3.38 mmol, 20.0 eq.) and $Pd(PPh_3)_4$ (9.8 mg, 8.4 μmol, 0.05 eq.) were transferred into a pressure tube under inert atmosphere (Ar). Degassed EtOH (0.5 mL), and degassed, deionized water (0.5 mL) were added. Bis(4-(2-(2-ethylhexyl)-9,9-dimethyl-7-bromoacridin-10(9H)-yl)phenyl)methanone (**5**; 165 mg, 0.17 mmol, 1.0 eq.) was dissolved in degassed toluene (1.8 mL) and added to the mixture. The pressure tube was closed and the solution was stirred at 120 °C for 72 h. The solution was allowed to cool to r.t. and extracted with $CHCl_3$. The combined organic fractions were washed with water and HCl solution (5 mL, 2 M). The organic phase was dried over $MgSO_4$ and the solvent was evaporated under reduced pressure. The crude product was further purified by flash chromatography on flash silica gel (Hex:EtOH 50:1) and a subsequent preparative SEC in $CHCl_3$. The product was obtained as a yellow solid (88 mg, 0.09 mmol, 52%).

R_f = 0.30 (SiO_2, Hex:EtOAc = 50:1); ^{1}H NMR (700 MHz, CH_2Cl_2-d2, δ): 8.19-8.15 (m, 4H, Ar H), 7.71 (d, *J* = 2.1 Hz, 2H, Ar H), 7.59-7.55 (m, 4H, Ar H), 7.47 (d, *J* = 7.8 Hz, 4H, Ar H), 7.29 (d, *J* = 2.1 Hz, 2H, Ar H), 7.26-7.21 (m, 6H, Ar H), 6.82 (dd, *J* = 8.3, 2.0 Hz, 2H, Ar H), 6.45 (dd, *J* = 8.6, 3.6 Hz, 2H, Ar H), 6.32 (dd, *J* = 8.4, 3.4 Hz, 2H, Ar H), 2.51 (d, *J* = 7.0 Hz, 4H, CH_2), 2.37 (s, 6H, CH_3), 1.75 (s, 12H, CH_3), 1.53 (d, *J* = 12.1 Hz, 2H, CH), 1.35-1.20 (m, 16H, CH_2), 0.91-0.86 (m, 12H, CH_3); ^{13}C NMR (176 MHz, CH_2Cl_2-d2, δ): 195.16, 146.21, 140.36, 138.66, 138.57, 137.14, 136.78, 135.00, 133.94, 133.03, 131.66, 131.07, 130.84, 129.81, 127.53, 126.66, 125.14, 124.42, 115.24, 114.75, 41.63, 39.80, 36.63, 32.67, 31.57, 31.56, 29.23, 25.86, 23.53, 21.15, 14.33, 11.01; MALDI-MS (m/z): $[M-CH3]^+$ calcd. 985.6036; found 985.6100; $[M]^+$ calc. 1000.6271; found 1000.6333.

Synthesis of ***P(C2-MAc-BP)***

A modified procedure based on literature was carried out.[1] 1,2-Bis(4-(4,4,5,5-tetramethyl-1,3,2-dioxaborolan-2-yl)phenyl)ethane (**7**; 133.2 mg, 0.307 mmol, 1.0 eq.), Na_2CO_3 (650 mg, 6.13 mmol, 20 eq.) and $Pd(PPh_3)_4$ (18 mg, 15 μmol, 0.05 eq.) were transferred into a pressure tube under inert atmosphere (Ar). Degassed EtOH (0.5 mL), and degassed, deionized water (0.5 mL) were added. Bis(4 (2-(2-ethylhexyl)-9,9-dimethyl-7-bromoacridin-10(9H)-yl)phenyl)methanone (**5**; 300.3 mg, 0.307 mmol, 1.0 eq.) was dissolved in degassed toluene (1.8 mL) and added to the mixture. The pressure tube was closed and the solution was stirred

at 120 °C for 72 h. The solution was allowed to cool to r.t. and 4,4,5,5-tetramethyl-2-(p-tolyl)-2,1,3,2-dioxaborolane (**6**; 7 mg, 0.03 mmol, 0.1 eq.) and $Pd(PPh_3)_4$ (7 mg, 6 μmol, 0.02 eq.) were added in Ar counterflow for an endcapping reaction. The closed pressure tube was stirred at 120 °C for another 24 h. The mixture was cooled to r.t. and extracted with $CHCl_3$. The combined organic fractions were washed with water and HCl solution (5 mL, 2 M). The organic phase was dried over $MgSO_4$ and the solvent was evaporated under reduced pressure. The crude product was further purified by soxhlet extraction (1) methanol, 2) isopropanol, 3) hexane 4) $CHCl_3$) and a subsequent preparative SEC in $CHCl_3$. The product was obtained as a yellow solid (282 mg, 92 %).

^{1}H NMR (700 MHz, CH_2Cl_2-d2, δ): 8.23-8.09 (m, 4H, Ar H), 7.73 (s, 2H, Ar H), 7.60-7.54 (m, 4H, Ar H), 7.54-7.48 (m, 4H, Ar H), 7.33-7.19 (m, 8H, Ar H), 6.82 (d, J = 8.3 Hz, 2H, Ar H), 6.46 (d, J = 8.5 Hz, 2H Ar H), 6.33 (d, J = 8.3 Hz, 2H Ar H), 3.03-2.91 (m, 4H, CH_2), 2.56-2.43 (m, 4H, CH_2), 1.74 (s, 11H), 1.68 (s, 1H), 1.64 (s, 1H), 1.59-1.50 (m, 5H), 1.38-1.17 (m, 18H), 0.95-0.78 (m, 13H); 13C NMR (176 MHz, CH_2Cl_2-d2, δ): 195.16, 146.18, 140.70, 140.42, 139.13, 138.65, 137.15, 135.01, 133.83, 133.04, 131.10, 130.81, 129.27, 127.53, 126.75, 126.66, 125.18, 124.48, 115.24, 114.73, 41.62, 39.79, 37.77, 36.63, 32.67, 31.76, 31.57, 29.22, 25.85, 23.52, 14.34, 11.02; SEC (THF, UV detection, PS calibration): $\bar{M}_n$ = 6.6 kg mol^{-1}, $Đ$ = 1.86.

Synthesis of ***P(C6-MAc-BP)***

A modified procedure based on literature was carried out.[1] 1,6-Bis(4-(4,4,5,5-tetramethyl-1,3,2-dioxaborolan-2-yl)phenyl)hexane (**8**; 177 mg, 0.36 mmol, 1.0 eq.), Na_2CO_3 (766 mg, 7.22 mmol, 20 eq.) and $Pd(PPh_3)_4$ (21 mg, 19 μmol, 0.05 eq.) were transferred into a pressure tube under inert atmosphere (Ar). Degassed EtOH (0.5 mL), and degassed, deionized water (0.5 mL) were added. Bis(4 (2-(2-ethylhexyl)-9,9-dimethyl-7-bromoacridin-10(9H)-yl)phenyl)methanone (**5**; 353.5 mg, 0.361 mmol, 1.0 eq.) was dissolved in degassed toluene (1.8 mL) and added to the mixture. The pressure tube was closed and the solution was stirred at 120 °C for 72 h. The solution was allowed to cool to r.t. and 4,4,5,5-tetramethyl-2-(p-tolyl)-2,1,3,2-dioxaborolane (**6**; 8 mg, 0.04 mmol, 0.1 eq.) and $Pd(PPh_3)_4$ (8 mg, 7 μmol, 0.02 eq.) were added in Ar counterflow for an endcapping reaction. The closed pressure tube was stirred at 120 °C for another 24 h. The mixture was cooled to r.t. and extracted with $CHCl_3$. The combined organic fractions were washed with water and HCl solution (5 mL, 2 M). The organic phase was dried over $MgSO_4$ and the solvent was evaporated under reduced pressure. The crude product was further purified by soxhlet extraction (1) methanol, 2) isopropanol, 3)

hexane 4) $CHCl_3$) and a subsequent preparative SEC in $CHCl_3$. The desired product was obtained as a yellow solid (324 mg, 85 %).

^{1}H NMR (700 MHz, CH_2Cl_2-d2, δ): 8.19-8.12 (m, 4H, Ar H), 7.71 (s, 2H, Ar H), 7.60-7.52 (m, 4H, Ar H), 7.49 (d, *J* = 7.9 Hz, Ar H), 7.28 (s, 2H, Ar H), 7.26-7.16 (m, 6H, Ar H), 6.81 (d, *J* = 8.4 Hz, 2H, Ar H), 6.45 (d, *J* = 8.6 Hz, 2H, Ar H), 6.32 (d, *J* = 8.4 Hz, 2H, Ar H), 2.67-2.60 (m, 4H, CH_2), 2.54-2.44 (m, 4H, aliphatic), 1.74 (s, 12H, CH_3), 1.70-1.60 (m, 6H, CH_2), 1.58-1.47 (m, 6H, CH_3), 1.41 (s, 4H, aliphatic), 1.35-1.17 (m, 19H, aliphatic), 0.93 – 0.80 (m, 14H, -CH_2CH_3); ^{13}C NMR (176 MHz, CH_2Cl_2-d2, δ): 195.21, 146.20, 141.84, 140.35, 138.82, 138.66, 137.13, 134.99, 133.99, 133.03, 131.65, 131.06, 130.84, 129.19, 127.52, 127.23, 126.67, 125.17, 124.47, 115.24, 114.75, 41.62, 39.80, 36.63, 35.89, 32.67, 31.96, 31.56, 31.54, 29.62, 29.57, 29.22, 25.86, 23.52, 14.34, 11.02; SEC (THF, UV detection, PS calibration): $\overline{M}_n$ = 7.2 kg mol^{-1}, *Đ* = 1.84..

Synthesis of ***P(Ph-MAc-BP)***

A modified procedure based on literature was carried out.[1] 4,4'-Bis(4,4,5,5-tetramethyl-1,3,2 dioxaborolan-2-yl)-1,1'-biphenyl (**9**; 105.2 mg, 0.259 mmol, 1.0 eq.), Na_2CO_3 (550 mg, 5.18 mmol, 20 eq.) and $Pd(PPh_3)_4$ (15 mg, 13 μmol, 0.05 eq.) were transferred into a pressure tube under inert atmosphere (Ar). Degassed EtOH (0.5 mL), and degassed, deionized water (0.5 mL) were added. Bis(4-(2-(2-ethylhexyl)-9,9-dimethyl-7-bromoacridin-10(9H)-yl)phenyl)methanone (**5**; 253.5 mg, 0.259 mmol, 1.0 eq.) was dissolved in degassed toluene (1.8 mL) and added to the mixture. The pressure tube was closed and the solution was stirred at 120 °C for 72 h. The solution was allowed to cool to r.t. and 4,4,5,5-tetramethyl-2-(p-tolyl)-2,1,3,2-dioxaborolane (**6**; 6 mg, 0.03 mmol, 0.1 eq.) and $Pd(PPh_3)_4$ (6 mg, 5 μmol, 0.02 eq.) were added in Ar counterflow for an endcapping reaction. The closed pressure tube was stirred at 120 °C for another 24 h. The mixture was cooled to r.t. and extracted with $CHCl_3$. The combined organic fractions were washed with water and HCl solution (5 mL, 2 M). The organic phase was dried over $MgSO_4$ and the solvent was evaporated under reduced pressure. The crude product was further purified by soxhlet extraction (1) methanol, 2) isopropanol, 3) hexane 4) $CHCl_3$) and a subsequent preparative SEC in $CHCl_3$. The desired product was obtained as a yellow solid (156 mg, 62 %).

^{1}H NMR (700 MHz, CH_2Cl_2-d2, δ): 8.22-8.18 (m, 4H, Ar H), 7.80 (s, 2H, Ar H), 7.75-7.71 (m, 4H, Ar H), 7.71-7.67 (m, 4H, Ar H), 7.59 (d, *J* = 8.0 Hz, 4H, Ar H), 7.35-7.29 (m, 4H, Ar H), 6.83 (d, *J* = 8.5 Hz, 2H, Ar H), 6.49 (d, J = 8.6 Hz, 2H, Ar H), 6.34 (d, *J* = 8.2 Hz, 2H, Ar H), 2.54-2.48 (m, 4H, CH_2), 1.77 (s, 12H, CH_3), 1.68 (s, 1H, aliphatic), 1.64 (s, 1H, aliphatic),

1.59-1.51 (m, 6H, aliphatic), 1.34-1.21 (m, 20H, alihatic), 0.92-0.82 (m, 15H, aliphatic); ^{13}C NMR (176 MHz, CH_2Cl_2-d2, δ): 195.18, 146.13, 140.69, 140.39, 139.13, 138.60, 137.24, 135.09, 133.32, 133.08, 131.68, 131.21, 130.75, 127.53, 127.31, 127.20, 126.69, 125.26, 124.55, 115.27, 114.73, 41.63, 39.80, 36.65, 32.68, 31.64, 29.23, 25.86, 23.53, 14.34, 11.02; SEC (THF, UV detection, PS calibration): $\overline{M}_n$ = 7.3 kg mol^{-1}, $Đ$ = 1.83.

The proton NMR spectra of the TADF-polymers are displayed in Figure S1. The GPC traces and results are respectively depicted and listed in Figure S2 and Table S1.

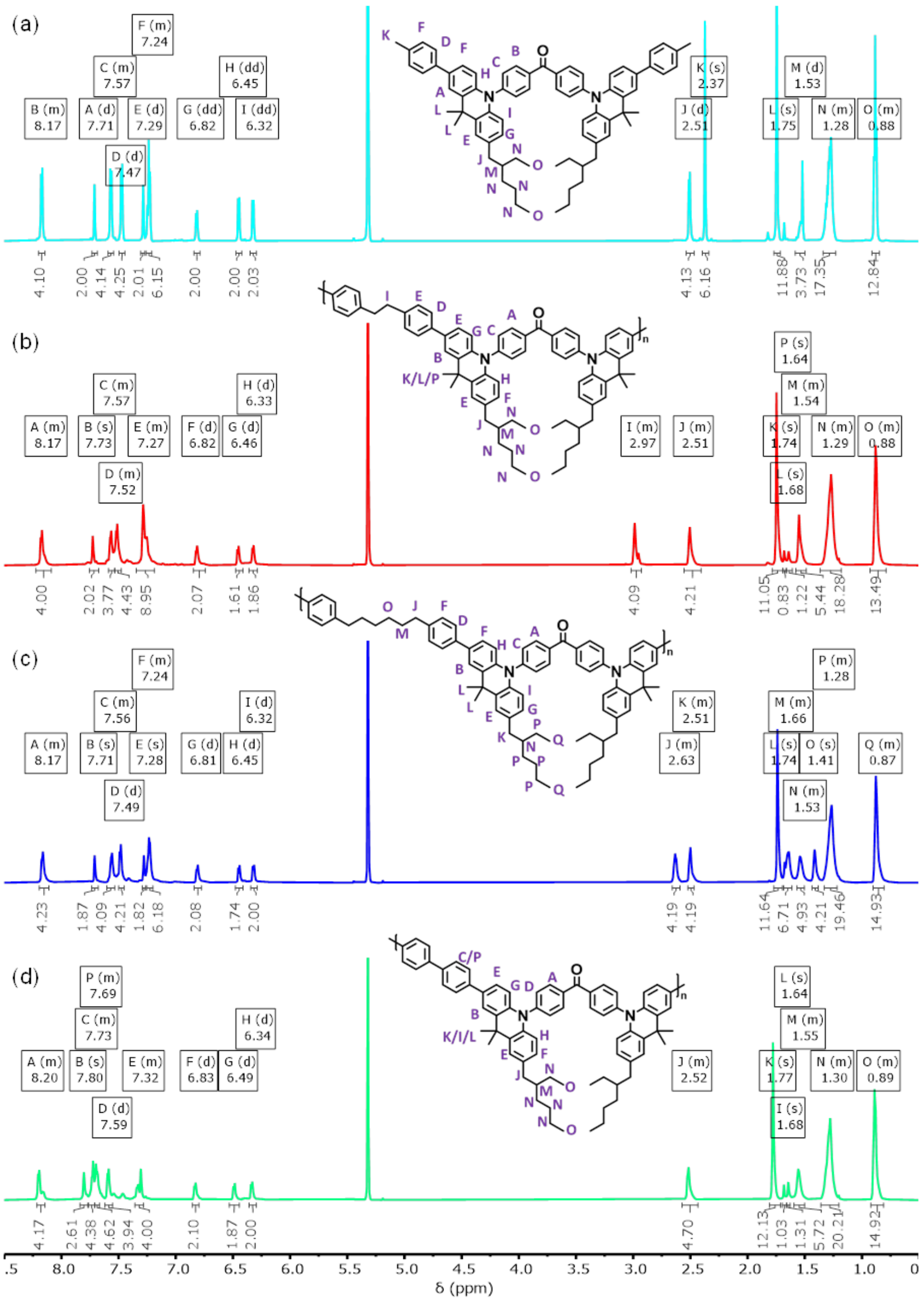

Figure S1. ^{1}H NMR spectra of (a) **Tol-MAc-BP**, (b) **P(C2-MAc-BP)**, (c) **P(C6-MAc-BP)** and (d) **P(Ph-MAc-BP)** in CH_2Cl_2-d2.

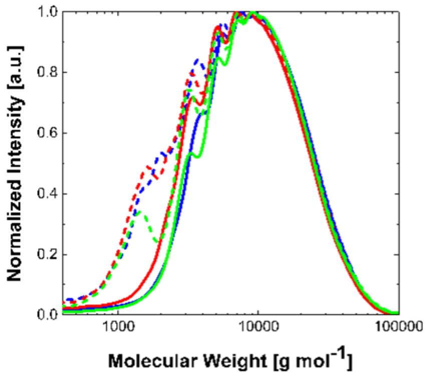

Figure S2. Molecular weight distribution after Soxhlet purification (dashed lines) and preparative SEC (solid lines) from SEC in THF calibrated via PS standard of **P(C2-MAc-BP)** (red), **P(C6-MAc-BP)** (blue) and **P(Ph-MAc-BP)** (green).

Table S1. GPC data of the TADF polymers after Soxhlet and SEC purification

Compound	$\bar{M}_n$ [kg mol^{-1}]	$\bar{M}_w$ [kg mol^{-1}]	$Đ$
**P(C2-MAc-BP)**	6.6	12.2	1.86
**P(C6-MAc-BP)**	7.2	13.3	1.84
**P(Ph-MAc-BP)**	7.3	13.3	1.83

The molecular structure of p-pTFF-C_2F_5SIS is depictured in Figure S3.

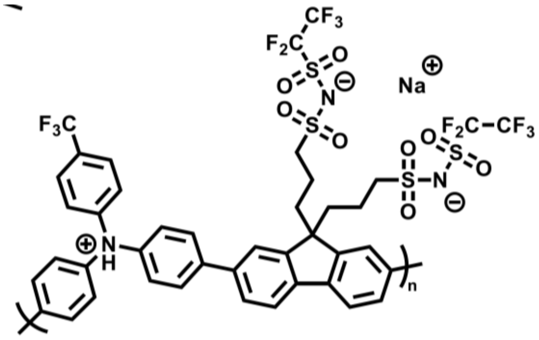

Figure S3. Molecular structure of poly(9,9-bis(3-(pentafluoroethanesulfonyl-imidosulfonyl)propyl)fluorine 2,7 diylalt 1,4 phenylene (p trifluoromethylphenylimino) 1,4 phenylene) (p-pTFF-C_2F_5SIS).

S2. Cyclic voltammetry and ultraviolet photoelectron spectroscopy

Cyclic voltammetry (CV) measurements were performed in dichloromethane solutions using Bu_4NPF_6 as a supporting electrolyte and ferrocene as an internal reference. The voltammograms are shown in Figure S4. The HOMO ebergy level E_{LUMO} is assumed to be approximately equal to the ionization potential (IP_{CV}). The latter was calculated by comparing the onset of oxidation of the analyte E_{ox} with that of the standard ($E_{HOMO,Fc}$) = -4.8 eV). This allows the estimation of the HOMO energy as: $E_{HOMO} \approx IP_{CV} = -e\left(\left(E_{ox} - E_{Fc/Fc^{+}}\right) - E_{HOMO,Fc}\right)$, with e the elementary charge. The LUMO or electron affinity (EA_{CV}) is obtained once a reduction peak has been measured: $E_{LUMO} = EA_{CV} = -e\left(\left(E_{red} - E_{Fc/Fc^{+}}\right) - E_{HOMO,Fc}\right)$. A reduction peak was only recorded for **Tol-MAc-BP** and **P(C6-MAc-BP)**. For the other materials the LUMO energy was estimated by adding energy of the optical bandgap (E_g) derived from the onset of the optical absorption spectrum (Figure S6) to the HOMO energy: $E_{LUMO} \approx E_{HOMO} + E_g$.

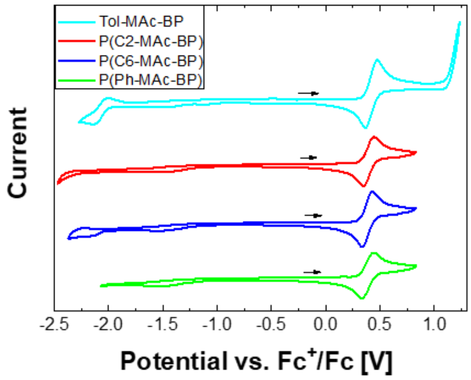

Figure S4. Cyclic voltammograms of **Tol-MAc-BP**, **P(C2-MAc-BP)**, **P(C6-MAc-BP)** and **P(Ph-MAc-BP)** in CH_2Cl_2 with 0.1 M Bu_4NPF_6 as supporting electrolyte. The ferrocene internal reference was added after the scan and the CV was remeasured.

The materials were further investigated by ultraviolet photoelectron spectroscopy (UPS). For this, thin films of the materials were spin-cast onto silicon wafers, covered with 2 nm of chromium and 50 nm of gold. The resulting UPS spectrum shows the intensity of the detected electrons plotted against the binding energy. For each of the samples, three UPS spectra were recorded for different spots of the film to increase accuracy. The spectra are given in Figure

S5. For the evaluation of the UPS ionization potential IP_{UPS}, the onsets on both ends of the spectra were obtained from the intersection of two linear fits (see Figure S5 b-c). At high binding energies close to the energy of the excitation light, the secondary electron cut-off E_{co} is obtained. The energies on the other end of the spectra give the HOMO edge ($E_{HOMO}^{(\text{edge})}$). With E_{co} and $E_{HOMO}^{(\text{edge})}$, the ionization potential was calculated using: $E_{HOMO} \approx IP_{UPS} = h\nu - \left(E_{co} - E_{HOMO}^{(\text{edge})}\right)$, with h Planck's constant and ν is the frequency of the excitation radiation. The LUMO was estimated through addition of the optical gap: $E_{LUMO} \approx IP_{UPS} + E_g$.

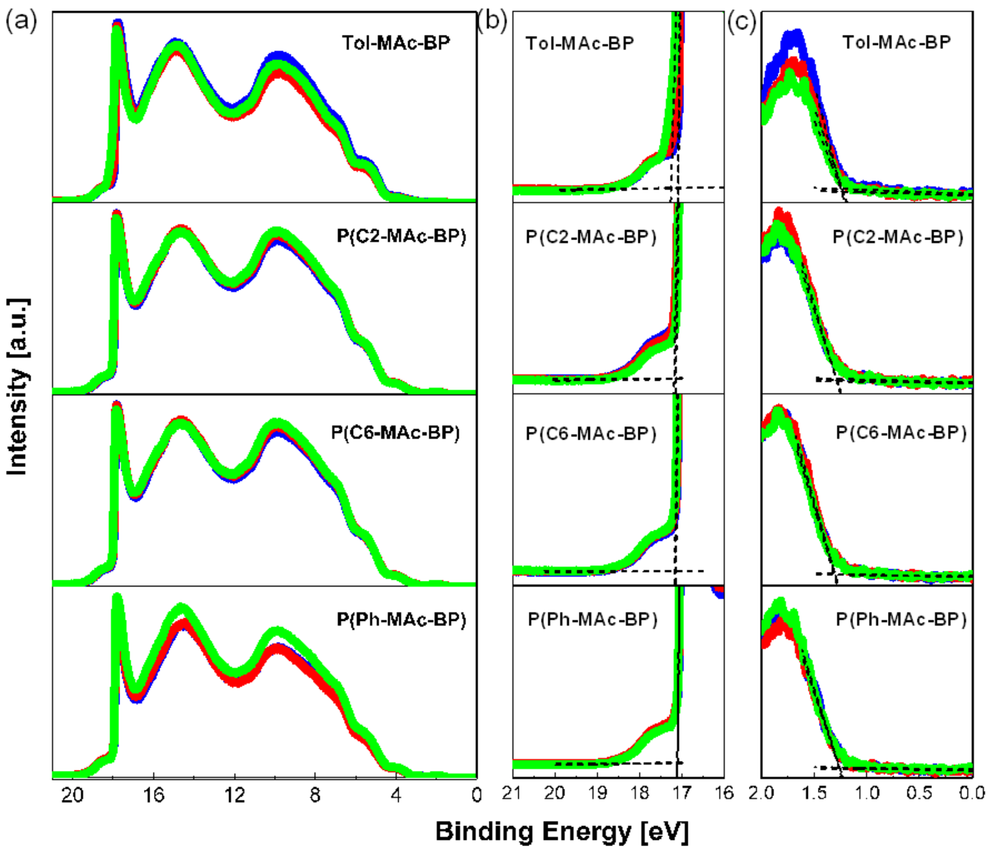

Figure S5. UPS spectra of the TADF materials prepared in this work.

S3. Spectroscopy

The normalized steady state optical absorption (UV-vis) and luminescence spectra of the TADF materials are combined in Figure S6a. The onset of the absorption spectrum gives an estimate for the optical gap E_g (S_1 excited state) being around 2.60, 2.61, 2.65 and 2.63 eV for **Tol-MAc-BP**, **P(C2-MAc-BP)**, **P(C6-MAc-BP)** and **P(Ph-MAc-BP)**, respectively. We do not suppose that the small differences in these values are physically relevant. Figure S6b displays the photoluminescence quantum yield (PLQY) of our TADF emitters in toluene

solution before and after nitrogen purging. The significant rise in PLQY upon exclusion of oxygen, observed for all materials, indicates involvement of the triplet excited state, as expected for TADF emitters. The PLQY was measured using an integrating sphere (F-3018 from Horiba Jobin Yvon) using a literature procedure.[2]

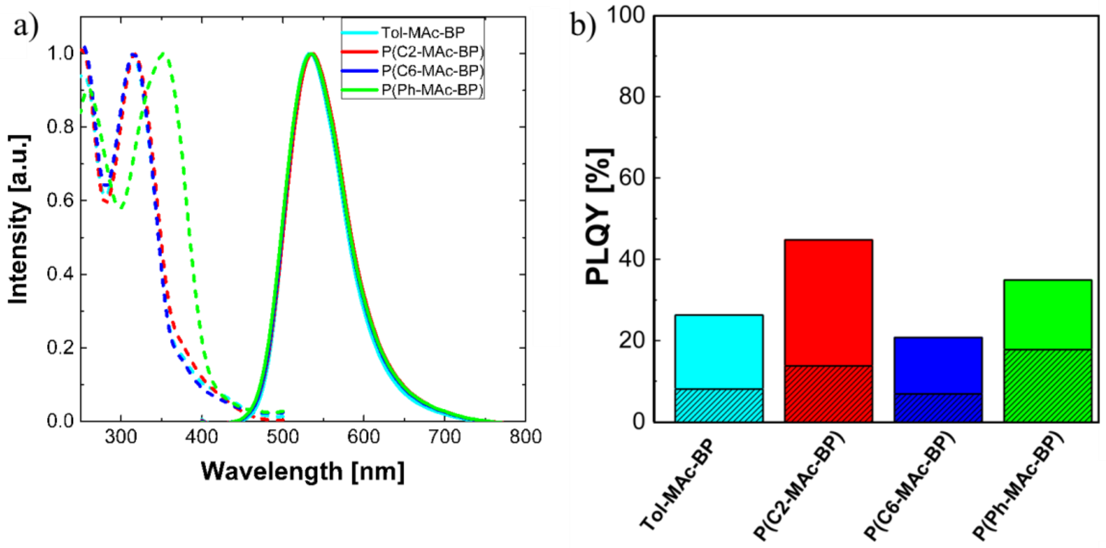

Figure S6. a) Steady state optical absorption (dashed) and luminescence spectra (solid) of films of the TADF materials prepared in this work. b) Photoluminescence quantum yield in toluene solution under ambient conditions (striped) and under nitrogen (non-striped).

Figure S7 displays the TRPL spectra (delayed fluorescence, DF) recorded as a function of excitation power, as well as the integrated signal plotted as a function of power, demonstrating a linear dependence. None of the TADF materials in the blended films showed any change in spectral line shape of the DF emission within the applied laser power range, demonstrating the absence of additional emissive species. Linear proportionalities the integrated DF and the excitation power for all materials across the full power range demonstrate a single photon upconversion process to responsible for the DF, proving TADF as its main origin. The decrease in PL intensity at high laser powers for all materials is attributed to irreversible bleaching of the sample.

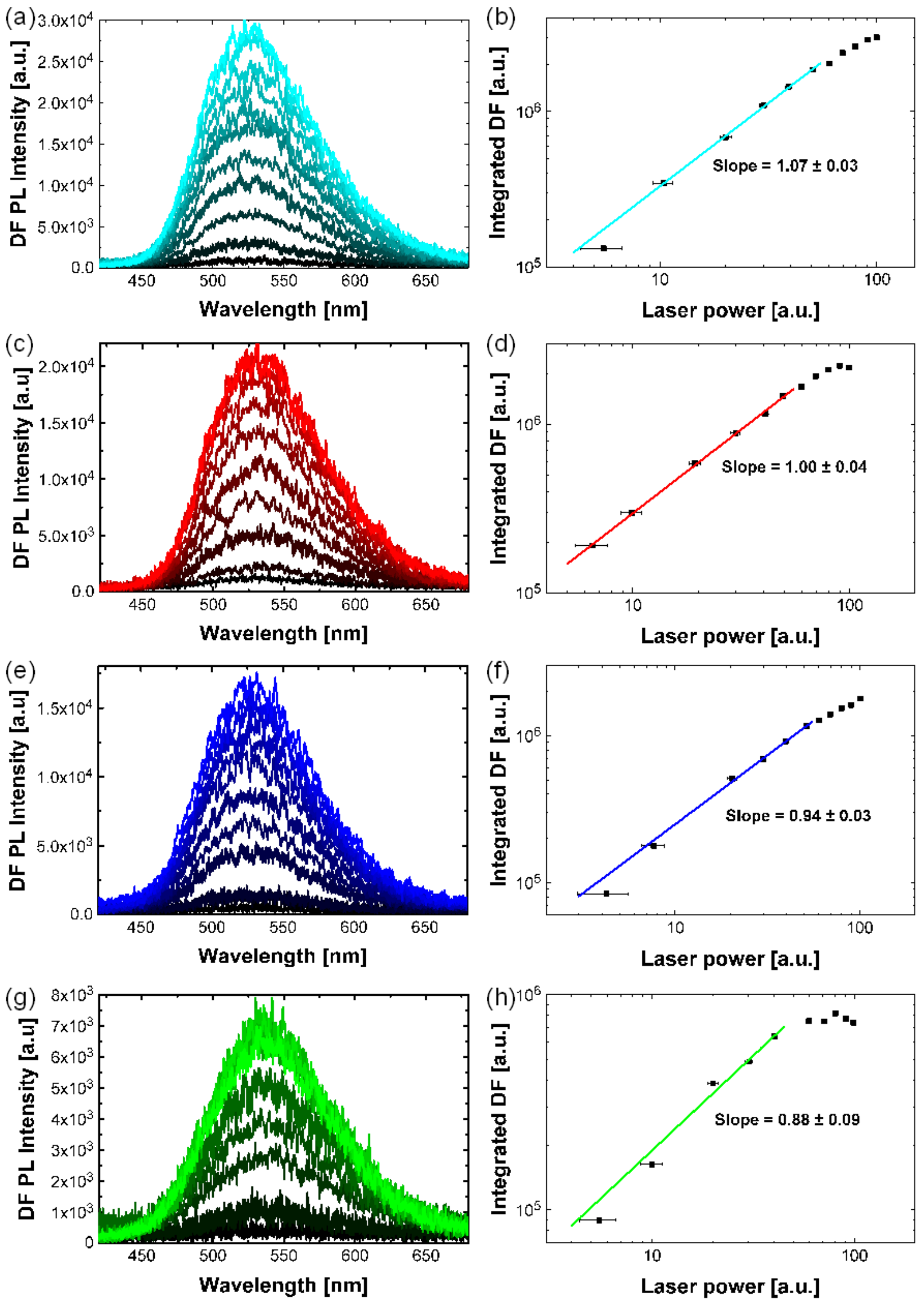

Figure S7. Power dependence of the delayed fluorescence in blended films of the TADF materials diluted 10 wt% in PS (λ_{exc} = 400 nm): DF spectra with increasing laser power and linear fit of log-log plot of DF intensity as a function of excitation power of (a, b) **Tol-MAc-BP** (black → cyan), (c, d) **P(C2-MAc-BP)** (black → red), (e, f) **P(C6-MAc-BP)** (black → blue) and (g, h) **P(Ph-MAc-BP)** (black → green). The laser power in (b), (d), (f) and (h) is plotted in arbitrary units.

S4 Devices

The current-voltage ($J - V$) behavior of hole only (HO) devices of the TADF materials is given as a function of temperature in Figure S8.

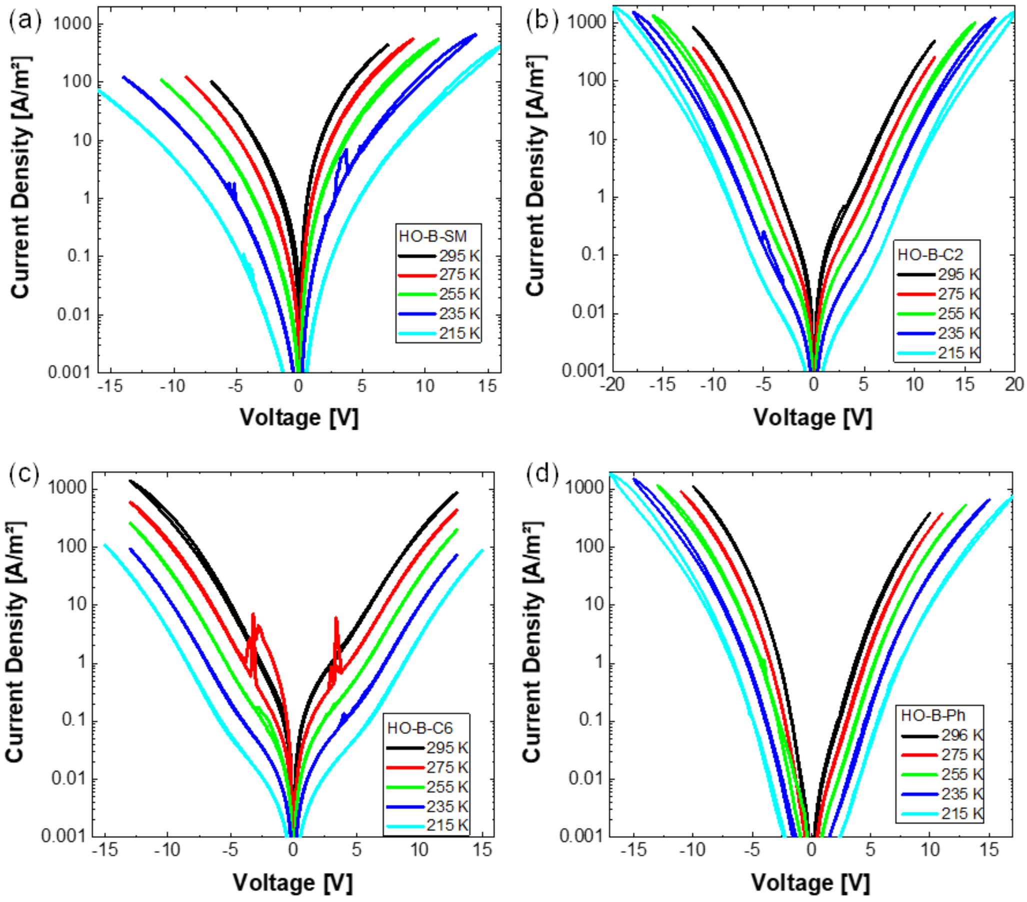

Figure S8. $J - V$ characteristics of the hole only devices (device structure in the Experimental Section of the main document) a) **Tol-MAc-BP** (191 nm), b) **P(C2-MAc-BP)** (77 nm), c) **P(C6-MAc-BP)** (76 nm) and d) **P(Ph-MAc-BP)** (77 nm) at various temperatures as indicated.

In Figure S9 the HO current-voltage data has been fitted by the drift-diffusion model mentioned in the main document, in order to quantify charge carrier mobility and trap density.

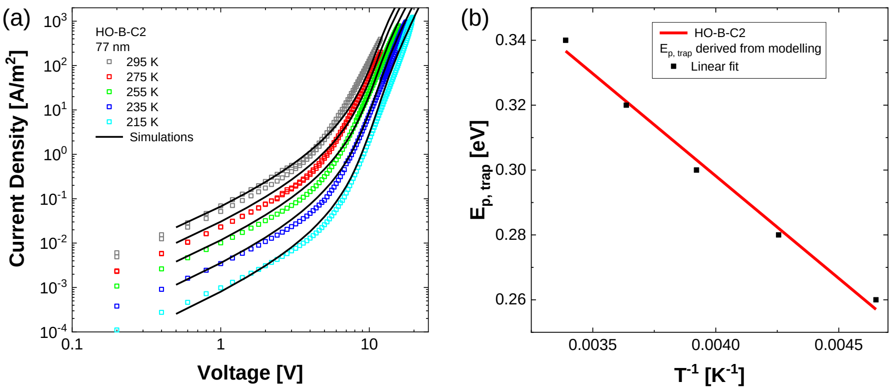

Figure S9. a) $J-V$ characteristics of the hole only device based on **P(C2-MAc-BP)** at various temperatures (symbols); the lines represent fits using the drift-diffusion model described in the main document. b) Trap level as a function of inverse temperature.

The current-voltage ($J-V$) behavior of electron only (EO) devices of the TADF materials is given for T = 295 K in Figure S10.

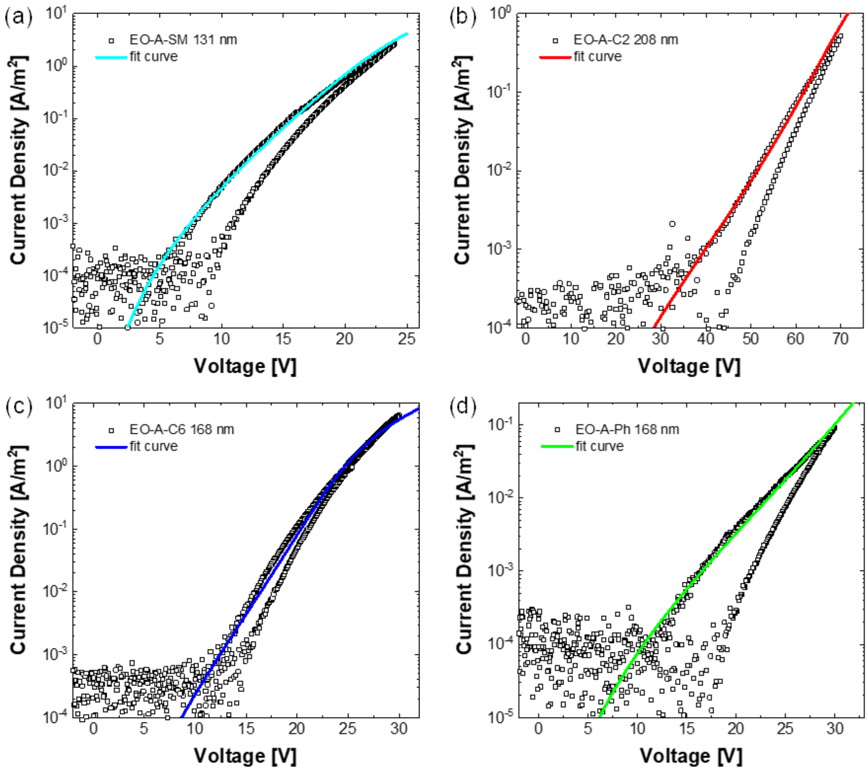

Figure S10. $J-V$ characteristics (symbols and lines respectively indicating measurements and fits) of electron-only devices of a) **Tol-MAc-BP**, b) **P(C2-MAc-BP)**, c) **P(C6-Mac-BP)** and d) **P(Ph-MAc-BP)**. Layer thicknesses are shown in the legends. T = 295 K.

The results obtained from curve fitting of the EO data is summarized in Table S2.

Table S2. Fit parameters of the electron-only devices EO-A.

Compound	μ_{n0} [10^{-12} $m^2V^{-1}s^{-1}$]	N_t [10^{23} m^{-3}]	$E_{n,Gauss}$ [eV]	$\sigma_{n,Gauss}$ [eV]
EO-A-SM	8	5.6	0.36	0.12
EO-A-C2	4	6.1	0.45	0.12
EO-A-C6	30	4.0	0.42	0.11
EO-A-Ph	9	5.1	0.42	0.11

The performance of the OLED (dual carrier) devices has been summarized in Table S3.

Table S3: Summary of device performance of OLED devices based on **Tol-MAc-BP** (A), **P(C2-MAc-BP)** (B), **P(C6-MAc-BP)** (C) and **P(Ph-MAc-BP)** (D), diluted in PS at the indicated weight percentages (first column).

Device	V_{on} [V] at 1 cd m^{-2}	V_d [V]	CE [cd A^{-1}]	PE [Lm W^{-1}]	EQE [%]	λ_{em} [nm]	FWHM [nm]	CIE (x, y)
		max./ 100 /1000 cd m^{-2}						
A-SM-pristine	3.0	3.0/ 3.6/ 4.5	15.5/ 12.9/ 10.8	16.2/ 11.5/ 7.7	7.5/ 6.3/ 5.3	528	91	0.34, 0.59
A-SM-50 %	3.0	3.5/ 3.8/ 4.8	23.1/ 22.9/ 20.4	22.2/ 19.3/ 13.5	12.6/ 12.5/ 11.1	522	86	0.32, 0.59
A-SM-25 %	3.1	3.7/ 4.2/ 5.3	23.4/ 23.4/ 22.8	22.8/ 17.3/ 11.9	12.9/ 12.6/ 11.0	520	86	0.31, 0.59
A-SM-10 %	4.3	5.3/ -/ -	3.9/ -/ -	2.4/ -/ -	2.4/ -/ -	513	81	0.27, 0.56
B-C2-pristine	3.1	3.2/ 4.6/ 6.9	13.1/ 10.7/ 6.0	12.8/ 7.4/ 2.7	6.7/ 5.5/ 3.1	545	100	0.39, 0.57

B-C2-50 %	3.4	3.7/ 5.0/ 7.0	18.8/ 16.5/ 10.8	16.8/ 10.5/ 4.9	9.7/ 8.5/ 5.6	535	96	0.37, 0.58
B-C2-25 %	3.6	4.5/ 5.0/ 6.7	18.9/ 18.6/ 13.2	14.4/ 11.7/ 6.2	9.9/ 9.8/ 6.9	530	93	0.35, 0.59
B-C2-10 %	4.2	5.0/ 6.7/ -	20.4/ 17.4/ -	13.9/ 8.21/ -	11.2/ 9.6/ -	523	89	0.32, 0.59
C-C6-pristine	3.2	3.5/ 4.5/ 6.0	13.9/ 12.6/ 9.7	12.5/ 8.9/ 5.2	7.1/ 6.5/ 5.0	537	94	0.37, 0.58
C-C6-50 %	3.5	4.1/ 5.0/ 6.4	18.1/ 14.9/ 14.1	15.0/ 9.5/ 7.0	9.7/ 8.0/ 7.5	526	92	0.34, 0.59
C-C6-25 %	3.7	4.6/ 5.2/ 6.7	21.3/ 16.2/ 16.9	16.3/ 9.9/ 7.9	11.8/ 9.1/ 9.4	520	87	0.32, 0.59
C-C6-10 %	4.3	5.3/ 6.7/ -	20.0/ 18.1/ -	13.2/ 8.6/ -	11.4/ 10.4/ -	518	85	0.30, 0.58
D-Ph-pristine	3.0	3.5/ 4.7/ -	5.5/ 4.7/ -	5.3/ 3.1/ -	2.9/ 2.5/ -	399, 548	105	0.39, 0.56
D-Ph-50 %	3.4	3.5/ 5.1/ 7.8	8.6/ 6.7/ 2.9	7.8/ 4.2/ 1.2	4.5/ 3.5/ 1.5	399, 542	101	0.37, 0.57
D-Ph-25 %	3.5	3.6/ 5.2/ -	11.3/ 7.8/ -	9.8/ 4.7/ -	6.1/ 4.2/ -	399, 534	98	0.35, 0.57
D-Ph-10 %	3.8	4.0/ 6.0/ -	11.4/ 7.5/ -	11.9/ 4.0/ -	8.4/ 4.2/ -	399, 528	94	0.34, 0.57

In order to investigate if the insulating polystyrene as a host has a negative impact on charge transport and hence OLED performance, we fabricated two series of devices using a conventional semiconducting host. For the first set, TCTA was used as a host (devices E), while the second set comprises a mixed host of TCTA and TAPC (devices F), which has been used in combination with TADF polymers in solution processing.[1, 3] For both sets of devices

the doping concentration of the TADF materials is 10 %, resulting for the latter series in a ratio of TADF:TCTA:TAPC = 10:65:25. The EMLs were spincoated from chlorobenzene solution and, due to the low solubility of TCTA and TAPC, the solutions had to be heated to 80 °C to fully dissolve the materials. The J-V-L characteristics and the external quantum yield (EQE) of these devices is depicted in Figure S11. Overall, the EQE of these devices is somewhat lower in comparison to the devices based on polystyrene as a host (see main document).

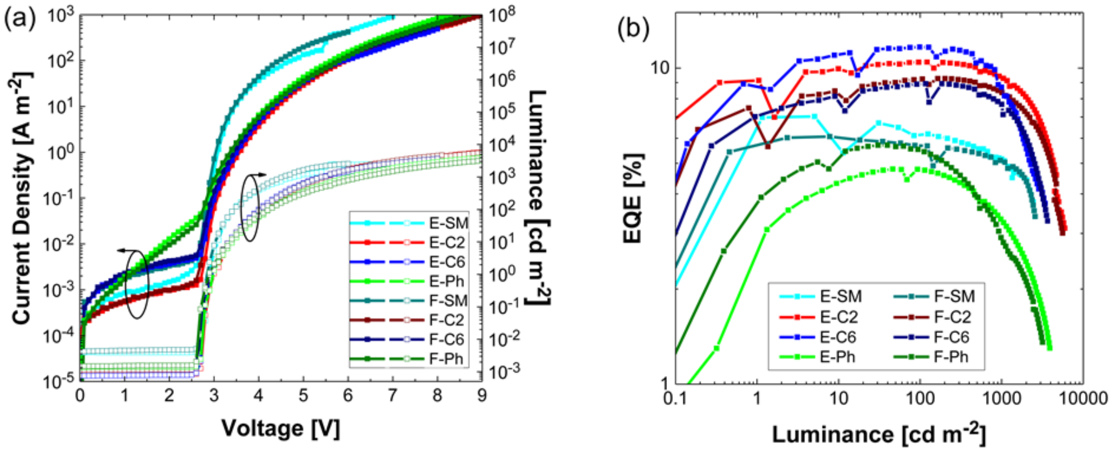

Figure S11. a) $J-V-L$ characteristics of devices based on Tol-MAc-BP (light blue), P(C2-MAc-BP) (red), P(C6-MAc-BP) (dark blue) and P(Ph-MAc-BP) (green) as emitter, blended 10% with TCTA ("E-series") and TCTA:TAPC (65:25) ("F-series"), as indicated in the legends. b) External quantum yield (EQE), plotted as a function of luminance of the same OLED devices (corresponding color code).

In Figure S12 we plot the normalized electroluminescence (EL) spectra of the OLED devices based on our TADF emitters, diluted in polystyrene at various ratios. The shape and position of the band is similar for all materials and consistent with the emission of the monomeric DMAC-BP chromophore. Interestingly, there is a small but clear blue-shift upon increasing the dilution. We suspect that this is a solvatochromic effect, known to occur for TADF emitters, on account of the charge transfer nature of their excited state.[4] The blue shift suggests that the environment of the chromophores becomes less polar upon increasing the dilution, which leads to a destabilization of the charge transfer state and hence a widening of the band gap.

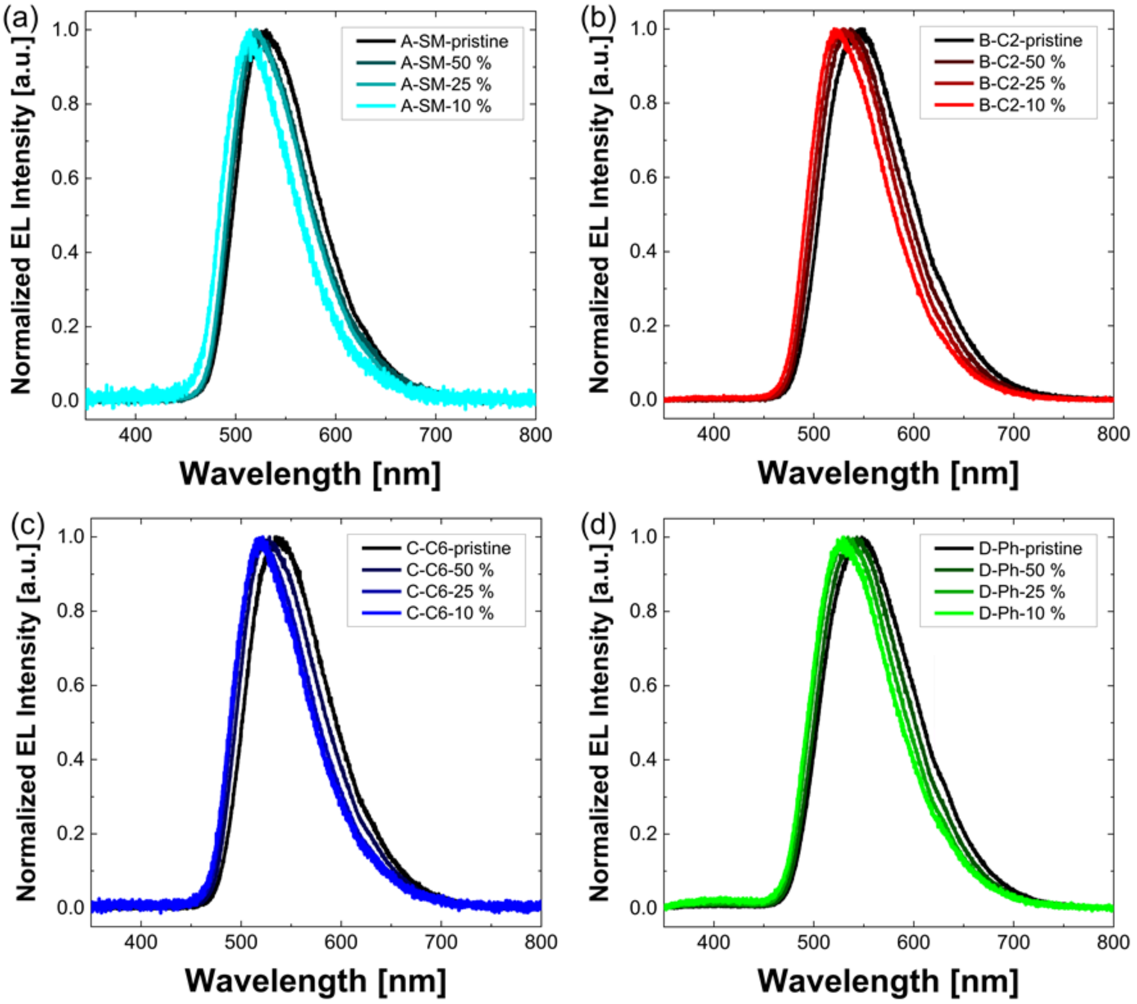

Figure S12. Electroluminescence spectra spectra of devices based on a) Tol-MAc-BP, b) P(C2-MAc-BP), c) P(C6-MAc-BP) and d) P(Ph-MAc-BP) with various polystyrene blending ratios.

S5. Blend film morphology

To check for possible phase separation of the TADF-polymer:polystyrene blends during solution casting, we recorded AFM images of dried spin-coated films. No signs of phase separation were detected, probably owing to the fact that we maintain a reasonably high mixing entropy by using a low molecular weight polystyrene (see Experimental Section), as explained in the main text. Figure S13 shows the AFM images for the films of **Tol-MAc-BP**:polystyrene and **P(C6-MAc-BP)**:polystyrene as representative results. No structuring was discerned and the films are smooth. As an illustrative comparison, Figure S13d reveals a blend of **P(C6-MAc-BP)** and the cyclo olefin copolymer Zeonex® 480 to exhibit phase separation during spincasting, clearly evidenced by the round dark domains, which represent

the emitter-rich phase. In contrast, the small molecular emitter **Tol-MAc-BP** does not phase separate, again likely owing to the higher mixing entropy, as explained in the main document.

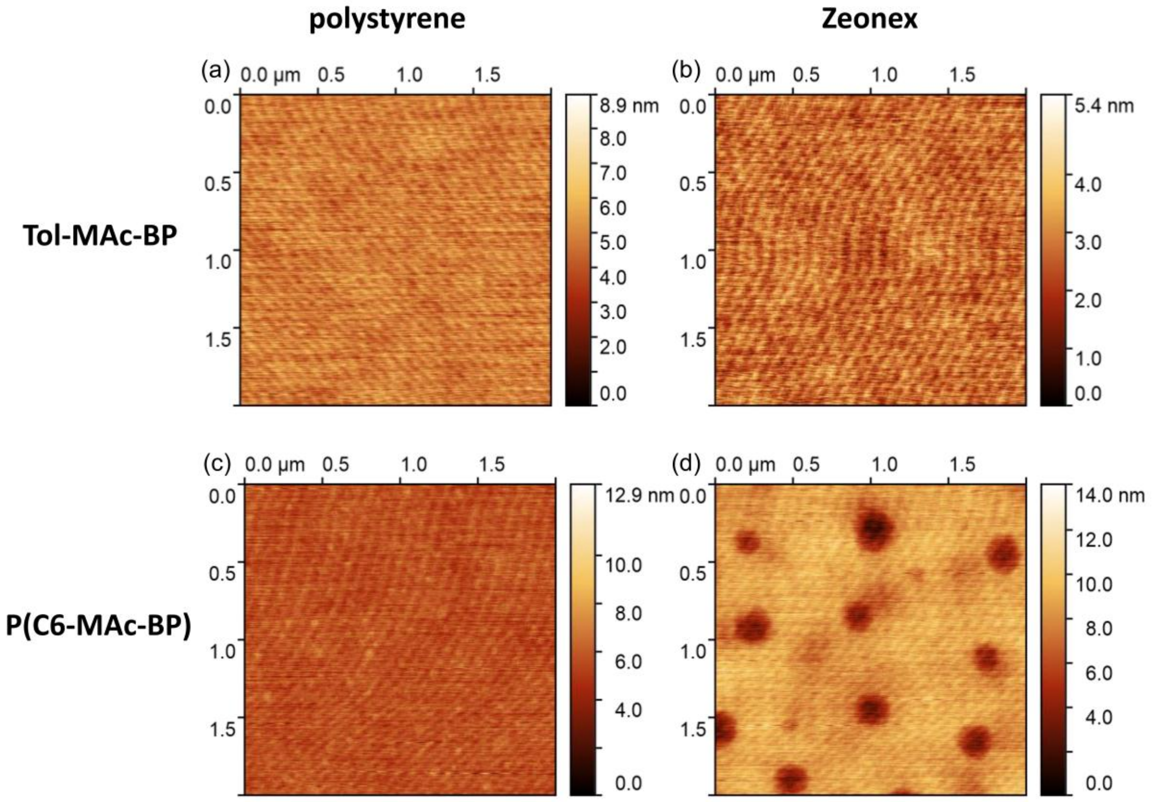

Figure S13. Topology AFM images (2 µm x 2 µm) of 2 wt% **Tol-MAc-BP** in a) low molecular weight polystyrene and b) Zeonex® 480, as well as **P(C6-MAc-BP)** in c) low molecular weight polystyrene and d) Zeonex® 480 host. The ribbon-like structure visible in all scans is a measurement artifact.

S5. References

[1] S. Y. Lee, T. Yasuda, H. Komiyama, J. Lee, C. Adachi, *Adv. Mater.* **2016**, *28*, 4019-4024.

[2]. L. Porrès, A. Holland, L. O. Pålsson, A. P. Monkman, C. Kemp, A. Beeby, J. Fluoresc. **2006**, *16*, 267–272.

[3] C. Li, R. S. Nobuyasu, Y. Wang, F. B. Dias, Z. Ren, M. R. Bryce, S. Yan, *Adv. Opt. Mater*. **2017**, *5*, 1700435.

[4] F. B. Dias, T. J. Penfold, A. P. Monkman, *Methods Appl. Fluoresc.* **2017**, *5*, 012001.
